# NAC regulation of embryo development in conifers

**DOI:** 10.1186/1753-6561-5-S7-P67

**Published:** 2011-09-13

**Authors:** Emma Larsson, Folke Sitbon, Jens Sundström, Sara von Arnold

**Affiliations:** 1 Swedish University of Agricultural Sciences, Department of Plant Biology and Forest Genetics, PO. Box 7080, 75007 Uppsala, Sweden

## Background

In most gymnosperms the cotyledons develop as a crown surrounding the incipient shoot apical meristem (SAM), which maintains the radial symmetry of the plant throughout embryogenesis. This is in contrast to the *Arabidopsis* embryo in which a symmetry-breaking event (from radial to bilateral symmetry) is associated with the emergence of the SAM and the two cotyledons [1]. We have previously shown that the differentiation of cotyledons and the establishment of the SAM and the root apical meristem (RAM) in somatic embryos of Norway spruce (*Picea abies*) is dependent on polar auxin transport (PAT) [2]. In *Arabidopsis* PAT restricts the expression of several genes belonging to the large plant specific NAC family of transcription factors [3-5], which in turn induce developmental processes involved in booth shoot and root formation. Members of the NAC family have a conserved DNA-binding domain in the N-terminal region known as the NAC domain, and a more diverse domain in the C-terminal region, proposed to be responsible for the trans-activation capacity of the protein [6]. Despite the diversity of the NAC gene family, and the importance of NAC proteins in fundamental processes, no NAC genes have to our knowledge been characterized in conifers.

## Methods

Phylogenetic analyses of NAC domain containing genes from *Picea glauca*, *Arabidopsisthaliana*, *Medicago truncatula* and *Physcomitrella patens* were performed using PAUP* and MrBayes. Full-length cDNAs of two NAC genes were isolated from maturing somatic embryos of Norway spruce and cloned using standard protocols. The promoters were amplified using the Genome Walker™ kit (Clontech). The motif discovery tool MEME was used to find conserved motifs in the C-terminal regions and in the promoters of the NAC sequences from different species. The expression pattern of the two isolated NAC genes was analyzed in Norway spruce somatic embryos using quantitative real-time PCR. Their expression was also analyzed in embryos that had been treated with the PAT inhibitor NPA as described earlier [2]. Promoter::GUS fusions were inserted into embryogenic cultures of Norway spruce using *Agrobacterium* mediated transformation.

## Results and discussion

We identified several different NAC sequences from white spruce in GenBank and deployed them to different sub-clades within the NAC gene family. Two full-length cDNAs (*gene 1* and *gene 2*) were cloned from Norway spruce*.* Both genes have elements in their promoter region which are significant for specific NAC genes. Furthermore, *gene 1* harbors previously characterized motifs that have been shown to have functional importance in NAC genes in *Arabidopsis*.

The relative expression of *gene 1* increased as early embryos differentiated, and remained at a high level until separated cotyledons were clearly visible. However, the up-regulation was blocked in embryos that formed fused cotyledons and lacked distinct SAM and RAM after being treated with NPA. The relative expression of *gene 2* was high in proembryogenic masses and early differentiating embryos but decreased as the embryos developed and matured. Interestingly the down-regulation occurred at about the same time as the cotyledons were delineated. The expression of *gene 2* was not dependent on PAT. Spatial expression analysis using promoter::GUS fusions revealed that the spruce genes have specific expression patterns, distinct from the expression patterns of their most closely related *Arabidopsis* counterparts.

## Conclusion

Taken together, our results show that NAC genes with distinct signature motifs existed before the separation of angiosperms and gymnosperms approximately 300 million years ago. However, through evolution the expression of closely related genes has diversified leading to induction of distinct developmental programs in angiosperms and gymnosperms.
